# Prenatal Hypoxia Triggers a Glucocorticoid-Associated Depressive-like Phenotype in Adult Rats, Accompanied by Reduced Anxiety in Response to Stress

**DOI:** 10.3390/ijms25115902

**Published:** 2024-05-28

**Authors:** Viktor Stratilov, Sofiya Potapova, Diana Safarova, Ekaterina Tyulkova, Oleg Vetrovoy

**Affiliations:** 1 Laboratory of Regulation of Brain Neuronal Functions, Pavlov Institute of Physiology, Russian Academy of Sciences, Makarova Emb. 6, 199034 Saint-Petersburg, Russia; 2 Department of Biochemistry, Faculty of Biology, Saint Petersburg State University, Universitetskaya Emb. 7–9, 199034 Saint-Petersburg, Russia

**Keywords:** prenatal hypoxia, maternal stress, glucocorticoid system, learned helplessness

## Abstract

Fetal hypoxia and maternal stress frequently culminate in neuropsychiatric afflictions in life. To replicate this condition, we employed a model of prenatal severe hypoxia (PSH) during days 14–16 of rat gestation. Subsequently, both control and PSH rats at 3 months old were subjected to episodes of inescapable stress to induce learned helplessness (LH). The results of the open field test revealed an inclination towards depressive-like behavior in PSH rats. Following LH episodes, control (but not PSH) rats displayed significant anxiety. LH induced an increase in glucocorticoid receptor (GR) levels in extrahypothalamic brain structures, with enhanced nuclear translocation in the hippocampus (HPC) observed both in control and PSH rats. However, only control rats showed an increase in GR nuclear translocation in the amygdala (AMG). The decreased GR levels in the HPC of PSH rats correlated with elevated levels of hypothalamic corticotropin-releasing hormone (CRH) compared with the controls. However, LH resulted in a reduction of the CRH levels in PSH rats, aligning them with those of control rats, without affecting the latter. This study presents evidence that PSH leads to depressive-like behavior in rats, associated with alterations in the glucocorticoid system. Notably, these impairments also contribute to increased resistance to severe stressors.

## 1. Introduction

The glucocorticoid and serotoninergic systems are widely acknowledged as exerting a pivotal influence on the development of anxiety and depression [1,2,3,4]. Modifications in glucocorticoid synthesis and release or alterations in the composition of serotonin receptors across various cell subtypes within limbic and thalamocortical brain regions inevitably impact mental states [5,6,7]. In addition to their hereditary and social origins, both anxiety and depression are intricately associated with disruptions during prenatal development that epigenetically affect both the serotoninergic and glucocorticoid systems [8,9,10]. Specifically, the widespread occurrence of these disorders can be attributed to the influence of environmental threats on both maternal physiology and fetal development.

A comprehensive analysis of clinical data and findings derived from animal studies reveals the intricate interplay between hypoxic placental pathologies and maternal stress. These factors often coexist in various prenatal disorders, collectively exerting a dual influence on the subsequent physical and cognitive development of the offspring [11,12,13]. Placental pathologies including pre-eclampsia, placental insufficiency, and placental ischemia, along with maternal cardiovascular diseases, lead to fetal hypoxia [14,15,16,17]. Over the course of postnatal development, fetal hypoxia leads to learning deficits, impaired memory function, and decreased attention capacity. As a result, it may substantially contribute to a wide range of neurological and neuropsychological disorders such as bipolar disorder and schizophrenia [18,19,20]. Another condition often comorbid with fetal hypoxia is prenatal stress, which impacts the fetus through the maternal glucocorticoid system. Rodent studies have yielded compelling evidence showing that prenatal stress can reprogram the functionality of the hypothalamic–pituitary–adrenal (HPA) axis and modulate the basal levels of corticosterone and circadian rhythms in the offspring [21,22]. Much evidence also suggests that prenatal stress may affect the development of a serotoninergic phenotype and subsequent serotonin production in the raphe nuclei [23,24].

While maternal stress may occur independently, both human [25] and feline [26] studies have shown that placental corticotropin-releasing hormone (CRH) released in a positive loop manner [27] may also influence the maternal glucocorticoid system during pregnancy, potentially resulting in glucocorticoid-related stress [28]. Epigenetic changes associated with prenatal stress increase risks of substance use disorder [29,30,31], specifically nicotine addiction [32,33]. Adult rats subjected to prenatal stresses also may exhibit an increased propensity for depressive [34,35,36] and anxiety-like behavior [37]. The response exhibited by animals with predisposition to depressive-like behavior to exceptionally stressful events is of significant interest. Our previous findings indicated a predisposition of the phenotype associated with prenatal severe hypoxia (PSH) to develop depression [35]. As suggested before, repeated exposure to stress increases susceptibility to affective disorders [38].

To replicate the co-occurrence of maternal stress and fetal hypoxia, we employed a model of PSH on the 14th–16th days of embryogenesis in rats. This timeframe corresponds to the 5th–8th weeks of human prenatal development, coinciding with the formation of the hippocampus (HPC) [39]. In this study, we induced a state of inescapable stress using the learned helplessness (LH) paradigm in adult control and PSH rats. Our aim was to investigate whether PSH led to a phenotype susceptible to anxiety-like behavior and to reveal the possible molecular basis for this behavior in extrahypothalamic areas such as the HPC, medial prefrontal cortex (PFC), and amygdala (AMG), as well as in the hypothalamus (HT).

## 2. Results

### 2.1. Impact of Prenatal Severe Hypoxia on Rat Behavior in Normal Adulthood and after Learned Helplessness

**Figure 1:**
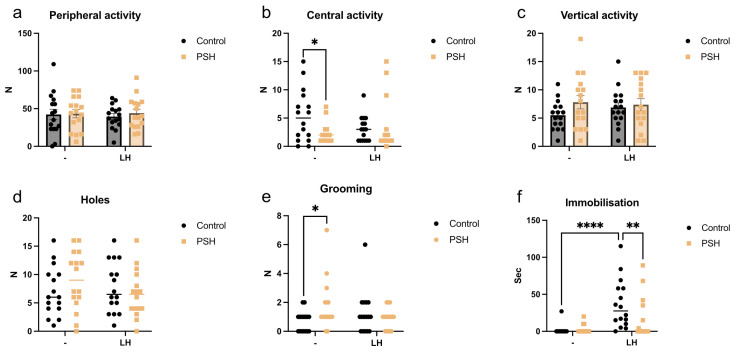
The effects of prenatal hypoxia and learned helplessness on the exploratory activity and anxious behavior of rats in the open field test: (**a**) number of crossed sections in the peripheral zone (peripheral activity); (**b**) number of crossed sections in the central zone (central activity), * intact PSH vs. intact control, Mann–Whitney’s test *p* = 0.036, *n* = 16 for each group; (**c**) number of rearings (vertical activity); (**d**) number of hole investigations (holes); (**e**) number of grooming episodes, * intact PSH vs. intact control, Mann–Whitney’s test, *p* = 0.022; (**f**) duration (s) of immobilization, **** intact control vs. control LH, Kruskal–Wallis *p* < 0.0001, Dunn test *p* < 0.0001, ** PSH LH vs. control LH, Dunn test *p* = 0.01, *n* = 16 for each group.

The comparative analysis revealed differences in the behavior of control and PSH rats, as assessed through the open field test (Figure 1). Intact PSH rats showed increased anxiety, which was expressed through reduced central activity (Figure 1b) and an increase in grooming activity (Figure 1e) compared with intact control animals. However, the behavior of PSH rats after LH episodes did not differ from the behavior of intact control animals. Meanwhile, LH increased immobilization in control animals compared with the intact control and even compared with PSH LH rats (Figure 1f). This indicates a diminished anxiety response in the PSH group following severe stress episodes, contrasting with the observed response in the control group.

### 2.2. Impact of Prenatal Hypoxia on the Expression of 5HT7R and Glucocorticoid Receptors in the Extrahypothalamic Brain Structures in Normal Adulthood and after Learned Helplessness

The analysis of 5-hydroxitriptamine receptor type 7 (5HT7R) and glucocorticoid receptor (GR) protein expression in the extrahypothalamic brain structures of control and PSH rats and in response to LH revealed some significant changes specific for each brain structure and condition studied.

**Figure 2:**
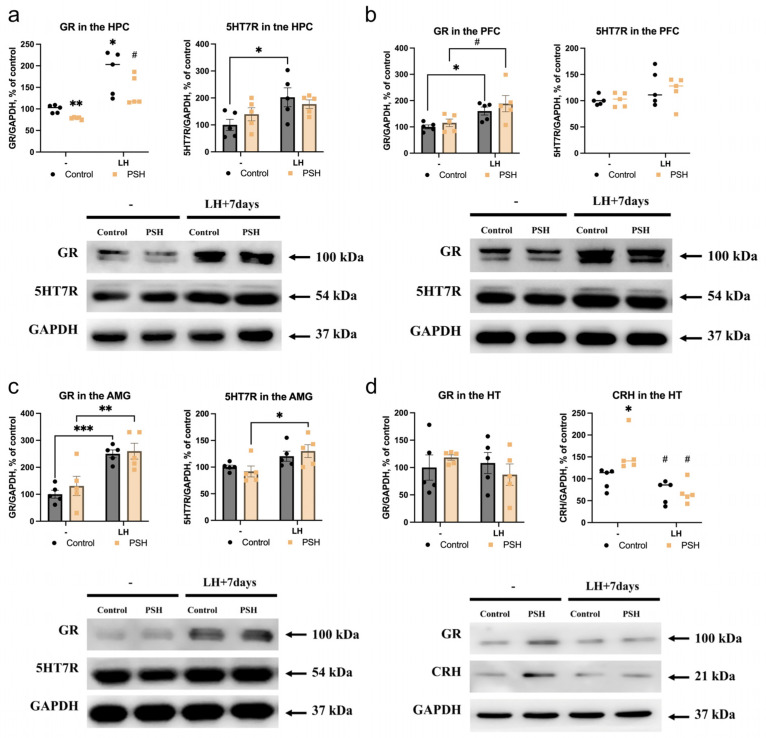
The effects of prenatal hypoxia and learned helplessness on the levels of GR and 5HT7R protein expression in the hippocampus (**a**), prefrontal cortex (**b**), amygdala (**c**), and on GR and CRH protein expression levels in the hypothalamus (**d**), and their respective representative Western blot images. (**a**) GR diagram: ** Welch ANOVA W (3, 7.06) = 17.66, *p* = 0.001; intact PSH vs. intact control, Welch’s *t*-test *p* = 0.004; * control LH vs. intact control, Welch’s *t*-test *p* = 0.02; ^#^ PSH LH vs. intact PSH, Welch’s *t*-test *p* = 0.015, *n* = 5. (**a**) 5HT7R diagram: * two-way ANOVA LH F (1, 16) = 7.833, *p* = 0.012; control LH vs. intact control, Fisher’s LSD test *p* = 0.01, *n* = 5. (**b**) GR diagram: * two-way ANOVA LH F (1, 16) = 12.14, *p* = 0.003; control LH vs. intact control, Fisher’s LSD test *p* = 0.04; ^#^ PSH LH vs. intact PSH, Fisher’s LSD test *p* = 0.016, *n* = 5. (**c**) GR diagram: *** two-way ANOVA LH F (1, 16) = 30.22, *p* < 0.0001; control LH vs. intact control, Fisher’s LSD test *p* = 0.0007. ** PSH LH vs. intact PSH, Fisher’s LSD test *p* = 0.002, *n* = 5. (**c**) 5HT7R diagram: * two-way ANOVA LH F (1, 16) = 9.8, *p* = 0.006; PSH LH vs. intact PSH, Fisher’s LSD test *p* = 0.01, *n* = 5. (**d**) CRH diagram: * Kruskal–Wallis, *p* = 0.004; intact control vs. intact PSH, Dunn test *p* = 0.009. ^#^ significant differences vs. intact PSH, intact PSH vs. PSH LH, Dunn test *p* = 0.009; control LH vs. intact PSH, Dunn test *p* = 0.008, *n* = 5.

In the hippocampus of intact PSH rats, we observed a decrease in GR protein levels (Figure 2a left) without changes in 5HT7R protein expression (Figure 2a right), in comparison with the intact control. In response to LH, in the HPC of both control and PSH rats, we found an increase in the amount of GR (Figure 2a left). However, after LH episodes, 5HT7R protein levels were increased in the control group only (Figure 2a right).

In the prefrontal cortex of intact PSH rats, we observed no changes in either GR (Figure 2b left) or 5HT7R (Figure 2b right) protein levels compared with the intact control. In response to LH, in the PFC of both the control and PSH groups, we found an increase in GR protein levels (Figure 2b left) compared with the intact control group and intact PSH (Figure 2b left), without any changes in 5HT7R protein expression (Figure 2b right).

In the amygdala of intact PSH rats, there were also no changes in GR (Figure 2c left) or 5HT7R (Figure 2c right) protein levels in comparison with the intact control group. In response to LH, in the AMG of both control and PSH rats, we found an increase in the amount of GR protein levels (Figure 2c left) compared with the intact control and PSH groups. In the PSH group, LH also increased the 5HT7R protein levels in the AMG (Figure 2c right).

### 2.3. Impact of Prenatal Hypoxia on the Hypothalamic Expression of Glucocorticoid Receptors and Corticotropin-Releasing Hormone in Normal Adulthood and after Learned Helplessness

In the hypothalamus, we found no differences in basal and LH-induced GR protein levels (Figure 2d left) between control and PSH animals. However, the CRH levels in the HT of intact PSH rats were heightened in comparison with the intact control (Figure 2d right). The LH episodes led to a decrease in the levels of CRH in the HT of PSH rats (Figure 2d right), reaching levels comparable to those in the control LH group (control LH vs. PSH LH, *p* = 0.917, Dunn test). However, the LH episodes had no effect on the levels of CRH in the HT of control rats (Figure 2d, right).

### 2.4. Impact of Prenatal Hypoxia on Glucocorticoid Receptor Nuclear Translocation in the Extrahypothalamic Brain Structures in Normal Adulthood and after Learned Helplessness

**Figure 3:**
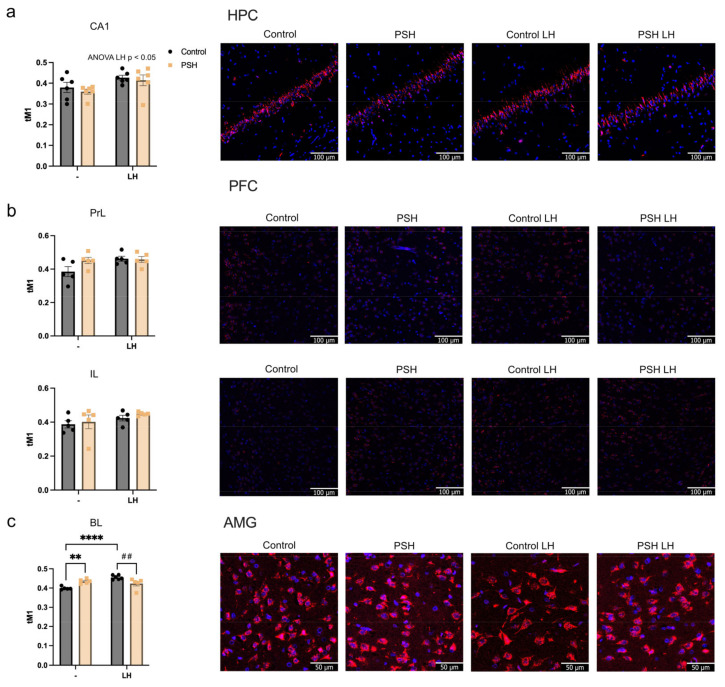
The effects of prenatal hypoxia and learned helplessness on GR protein nuclear translocation in the (**a**) CA1 area of the hippocampus, (**b**) prelimbic and infralimbic areas of the PFC, and (**c**) basolateral amygdala, detected via immunofluorescence. (**a**) two-way ANOVA LH F (1, 20) = 6781, *p* = 0.017. (**c**) ** intact control vs. intact PSH, *p* = 0.003, Tukey’s test; **** two-way ANOVA prenatal exposure x LH, F (1, 17) = 22.59, *p* = 0.0002; intact control vs. control LH, Tukey’s test *p* < 0.0001; ^##^ control LH vs. PSH LH, Tukey’s test *p* = 0.005, *n* = 5–6. Respective representative microphotographs showing GR protein nuclear translocation are provided, with red indicating GR-positive areas and blue representing DAPI-positive nuclei. Scale bars are 100 μm (**a**,**b**) and 50 μm (**c**).

The levels of GR nuclear translocation, assessed by colocalization of the immunofluorescence signal, demonstrated that in the CA1 area of the HPC, learned helplessness led to an increased Manders coefficient in both groups, without significant differences between them (Figure 3a).

In prelimbic and infralimbic parts of the PFC, we found no changes in the GR translocation either between intact groups or in response to LH (Figure 3b).

In the basolateral AMG we found an increased basal level of GR translocation in the intact PSH group compared with the intact control group (Figure 3c). After the LH episode, only control rats demonstrated an increase in GR nuclear translocation (Figure 3c), reaching levels even higher than those of the PSH group after LH (Figure 3c).

## 3. Discussion

In the current study, the PSH rats displayed depressive-like behavior under basal conditions, characterized by comparatively diminished central activity and increased grooming time in the open field test. The PSH-associated phenotype also exhibited elevated levels of hypothalamic CRH, which plays a central role in regulating the HPA axis. Taken together, these alterations reflect dysregulation in the HPA axis and possibly explain the depressive behavior observed in PSH rats during the open field test. The observed manifestations of the PSH-related depressive phenotype may be attributed to epigenetic modifications in DNA, such as CRH and GR promoter methylation, which can persist into adulthood and even have transgenerational effects [40,41]. In our prior study, we demonstrated that PSH induces a decrease in the glucocorticoid-dependent expression of genes related to glutamate metabolism. This led to glutamate insufficiency within the hippocampus, which is known to be a negative regulator of the hypothalamus’s production of CRH [22,42]. Consequently, this resulted in sustained hyperactivation of the HPA axis in PSH rats throughout their lifespan [22]. While hippocampal and PFC neurons are known to exert an inhibitory effect on HPA axis activation (including GR-dependent negative feedback regulation of the HPA axis), the amygdala, specifically the basolateral amygdala, plays a role in positive regulation of the HPA axis [43,44,45]. Stimulation of the amygdala by glucocorticoids via a feed-forward mechanism also induces CRH release in the hypothalamus [46] and may also modulate fear conditioning [47]. In our experiments, under basal conditions, the PSH group exhibited decreased hippocampal GR levels alongside increased GR nuclear translocation in the basolateral amygdala. This resulted in increased levels of CRH in the hypothalamus of PSH rats, disrupting their stress-response system [22,35] and contributing to their depressive phenotype.

The stress-induced LH consisting of three sessions had no discernible effect on the behavior of the PSH group in the open field test. In contrast, the control group exhibited clear signs of anxiety following three sessions of LH, as evidenced by an increase in their immobilization time. Remarkably, the central activity and grooming time of the PSH group after three LH sessions were similar to those in the intact control group. Following three LH sessions, both the control and PSH groups displayed evidence of HPA axis activation, as indicated by GR overexpression in the hippocampus, prefrontal cortex, and amygdala. Moreover, hippocampal GR translocation also increased in both groups. However, the control group exhibited a more pronounced increase in nuclear GR translocation in the amygdala following LH sessions compared with the PSH groups, which was consistent with the behavioral outcomes indicating increased anxiety in those animals.

It is widely accepted that the stimulation of hippocampal neurons by serotonin released from nucleus raphe projections [48] plays a role in maintaining stable expression of glucocorticoid receptors via 5HT7R [49]. Concurrently, persistent corticosterone stimulation may disrupt 5HT7R modulation in the raphe nuclei [50]. Among the various functions of 5HT7R, its activation leads to increased firing of glutamatergic hippocampal neurons, along with spontaneous activation of GABA interneurons [51], resulting in an overall excitatory effect in rodents. In the current study, only the control group demonstrated increased levels of hippocampal 5HT7R after LH episodes, while the effects of 5HT7R activation in the amygdala were somewhat unclear. However, it has been proposed that 5HT7R may be localized presynaptically within dopaminergic and serotoninergic projections to the amygdala, where it regulates dopamine and serotonin turnover [52]. Notably, while there were no differences in 5HT7R levels observed in the hippocampus and amygdala between intact control and PSH rats, following the LH episodes, only the control animals showed increased 5HT7R protein expression in the hippocampus, and only the PSH rats displayed this in the amygdala. This does not allow us to make any certain conclusion about the role of serotoninergic transmission in the changes detected in expression and translocation of GR.

Therefore, in this study, we have illustrated that intact PSH rats displayed a depressive-like phenotype. Conversely, the control but not the PSH group exhibited distinctly anxious behavior following the LH episodes. Our previous study speculated about the inability of PSH rats to maintain an adequate glucocorticoid feedback loop. This was supported by combination of elevated levels of CRH and corticosterone in their blood plasma with low hippocampal GR levels [22,35]. However, following the severe stress induced by the three LH sessions, the PSH group demonstrated functioning of the glucocorticoid feedback loop and resilience against anxious behavior, a response not typically observed in normal rats. Apart from the absence of evidence of anxious behavior following the LH episodes in PSH rats, the lack of anxiety was also reflected in a decrease in the initially increased CRH levels in the hypothalamus, bringing them down to the levels observed in the intact control rats.

Further research is essential to evaluate the occurrence of the observed phenomena across different mammal species and age groups. Nevertheless, these observations might reinforce the concept of “developmental programming through stress” [53]. These phenomena likely hold significant evolutionary value, potentially aiding in the preparation of individuals for survival in harsh conditions, by increasing their chances of reproduction, probably due to diminished reaction to stress.

## 4. Materials and Methods

### 4.1. Animals

Wistar rats from the Collective Usage Center’s biocollection of laboratory mammals of different taxonomic affiliation held at the Pavlov Institute of Physiology of RAS were used in the experiments. All experimental procedures were performed in compliance with the guidelines for reporting animal research [54] and approved by the Ethical Committee for the Use of Animal Subjects at the Pavlov Institute of Physiology. During the experiments, the researchers were blinded to the allocation of the groups. Animals were identified using random numbers, which were revealed only during data analysis after completion of the experiments.

### 4.2. Prenatal Severe Hypoxia

**Figure 4:**
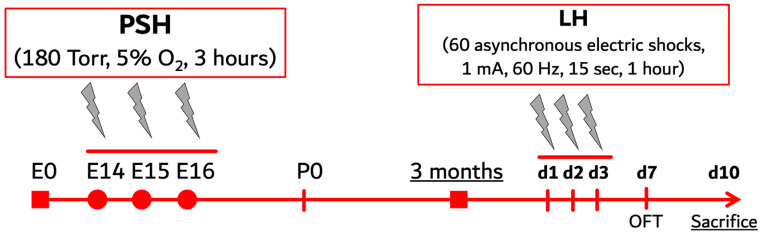
Scheme outlining the experimental study design and preparation of experimental groups. Four groups were prepared for the experiments: intact control, intact PSH (prenatal severe hypoxia), control exposed to three sessions of learned helplessness (control LH) at the age of 3 months, and PSH exposed to three sessions of LH (PSH LH) at the age of 3 months; E0–16, embryonic days; P0, day of birth; d1–10, days of the experiments with adult animals; OFT, open field test.

Prenatal severe hypoxia (PSH), discussed in our previous studies [33,35,42], was used as a reliable model of maternal stress response during pregnancy. The animals used in the experiments were born either from naive females (control) or females exposed to 3 sessions of severe hypobaric hypoxia on the 14th, 15th, and 16th (E14–16) days of pregnancy (PSH group) (Figure 4).

To produce hypoxia for obtaining the PSH group, we used a flow-type hypobaric chamber at a temperature of 20° to 25 °C in which the atmospheric pressure was gradually reduced to 180 Torr (5% of normobaric oxygen equivalent to 11,000 m above sea level) over 20 min. Pregnant dams were treated under such conditions for 3 h for three consecutive days on E14-E16 with an interval of 24 h between the sessions. The mortality rate in the hypobaric chamber was around 15%. Control females were also placed in the hypobaric chamber for 3 h daily on E14–16 days of pregnancy under normal atmospheric pressure and oxygen content.

Pups were weaned from their mothers at the age of 30 days. After weaning, the rats were housed in cages of 60 × 30 × 20 cm in size, with 6 animals in each. Each rat group consisted of randomly selected rats born from different dams to minimize litter bias. Rats received food and water ad libitum and were kept on a 12:12 h dark–light cycle at room temperature with a constant humidity of approximately 60%. In further experiments, adult male offspring with active spermatogenesis from the control and PSH groups were used. Each group consisted of 32 rats at the age of 3 months. All animal experiments were performed between 9 and 11 a.m.

### 4.3. Learned Helplessness Paradigm

This method was based on the paradigm commonly used to induce a mental state of learned helplessness to provide a reliable animal model of depression [55,56]. In order to induce learned helplessness (LH), 16 control and 16 PSH rats, aged 3 months, were exposed to inescapable treatment with 60 asynchronous electric shocks for 1 h (1 mA, 60 Hz, 15 s) in a chamber measuring 30 × 20 × 30 cm with a conductive floor (Figure 4). Animals were treated under these conditions for 3 consecutive days (d1–d3, Figure 4) with an interval of 24 h between the sessions. Intact control and intact PSH rats were also placed in the chamber for 1 h, but without electrical stimulation.

### 4.4. Open Field Test

The exploratory activity and anxious behavior of intact control and PSH rats or those stressed with LH was assessed in the open field test. Experiments were carried out in a location screened from incidental noise. Rats were tested four days after the LH (d7, Figure 4).

The open field test was performed in a round arena (diameter 97 cm, wall height 42 cm). The surface of the arena in the peripheral zone was divided into twelve sections and the central zone into seven sections. The central zone was under bright illumination (100–120 lux), while the peripheral zone was dimmed (40–50 lux).

Each rat was placed in the center of the arena. A ceiling-mounted camera recorded the movements of rats. The total number of sections crossed in the peripheral zone (peripheral activity) and the central zone (central activity), the number of rears (vertical activity), hole investigations (holes), and grooming activities, as well as the time of immobilization, were measured for a duration of 5 min [57].

### 4.5. Sample Preparation

To collect the brain samples for Western blotting and immunofluorescent, 3-month-old rats from all groups were sacrificed under isoflurane anesthesia using a guillotine, seven days after the last LH or sham procedure (d10, Figure 4). Following decapitation, tissues of the hippocampus (HPC), medial prefrontal cortex (PFC), amygdala (AMG), and hypothalamus (HT) were dissected and frozen in liquid nitrogen for later biochemical analysis. For immunofluorescence, brain samples containing the HPC, PFC, and AMG were submerged in a fixative solution (28 mL Fine Fix + 72 mL 96% ethanol, Milestone, Milan, Italy) for 24 h following a standard histological protocol. The tissues were dehydrated with 70% isopropanol for 1.5 h, followed by immersion in 80% isopropanol for another 1.5 h, and finally in 100% isopropanol for 3 h at room temperature. The samples were consecutively immersed in solutions containing isopropanol and mineral oil in ratios of 1:1, 1:2, and 1:5 for 1 h at every step. Following this, the samples were submerged in liquid paraffin twice, each time for one hour at 56 °C, before being sectioned. The 7 µm thick coronal sections of tissue samples were prepared using a rotary microtome (Sakura Accu-Cut, Sakura Seiki Co., Nagano, Japan). Sections were mounted onto poly-L-lysine covered slides, deparaffinized in xylol (twice for 5 min) and rehydrated in alcohol (96%→96%→96%→70% for 5 min in each solution).

### 4.6. Western Blotting

To obtain total protein extracts for Western blotting, the HPC, PFC, AMG, and HT tissues were homogenized in 50 mM Tris–HCl (pH 8.0) containing 150 mM NaCl, 1% Triton ×100 and a cocktail of protease and phosphatase inhibitors (SB-G2006, SB-G2007, Servicebio, Wuhan, China). Homogenates were incubated in a shaker for 30 min at +4 °C, centrifuged for 10 min at 14,000× *g*, and the supernatants were collected. Total protein content in the samples was measured using a PierceTM Rapid Gold BCA Protein Assay Kit (Thermo Scientific, Waltham, MA, USA) following the manufacturer’s protocol. Samples containing equal amounts of total protein were boiled for 10 min at +70 °C with a 3x Laemmli buffer.

The proteins in the samples were separated using SDS-PAGE (sodium dodecyl sulfate-polyacrylamide gel electrophoresis) and transferred to PVDF membranes (Thermo Scientific, USA). After blocking for 1 h in PBS with 5% skimmed milk, the membranes were incubated in PBS with rabbit anti-GR (1:2000, AF5004, Affinity Biosciences, Cincinnati, OH, USA), anti-5HT7R (1:2000, ab128892, Abcam, Cambridge, UK), anti-CRH (1:1000, ab184238, Abcam, UK), and anti-GAPDH (1:5000, AF7021, Affinity Biosciences, USA) primary antibodies for 2 h at room temperature.

The membranes were then washed three times with PBST (PBS with 0.1% Tween 20) and incubated in PBS with HRP-conjugated anti-rabbit (1:5000, E-AB-1003, Elabscience, Houston, TX, USA) secondary antibodies for 1 h at room temperature. The membranes were then washed twice with PBST. Immunoreactive protein bands were visualized via a Clarity ECL chemiluminescence kit (Bio-Rad, Hercules, CA, USA) with a ChemiScope 6000 Imaging System (Clinx Science Instruments, Shanghai, China).

The protein levels were quantified using ImageJ software (NIH, Bethesda, MD, USA) and normalized to GAPDH.

### 4.7. Immunofluorescent Analysis

For all structures, we also performed immunofluorescence staining to identify the level of GR nuclear translocation into DAPI-positive nuclei. For this, brain sections containing the CA1 area of the HPC (Bregma −3.24 mm), prelimbic (PrL) and infralimbic (IL) areas of the PFC (Bregma 2.76 mm), and also the basolateral (BL) AMG (Bregma −3.24 mm) were incubated overnight at 4 °C with primary rabbit polyclonal anti-GR (1:200, DF13247, Affinity Biosciences, USA) antibodies. After incubation with the primary antibodies and washing, all sections were incubated with secondary anti-rabbit AF647 (1:150, E-AB-1075, Elabscience Biotechnology, Houston, TX, USA) antibodies for 30 min. Afterwards, the sections were then incubated with DAPI reagent (E-IR-R103, Elabscience Biotechnology, USA) for 15 min to stain the nuclei. The sections were then washed with tap water, dried in ethanol, covered with permanent mounting media (Sub-X Mounting Medium; Leica Biosystems, Vista, CA, USA), and analyzed using an LSM 710 Carl Zeiss confocal microscope. The GR colocalization with DAPI was measured as the tM1 Manders coefficient, using ImageJ 1.54 software.

### 4.8. Statistical Analysis

Statistical analysis was performed using Prism 10 (GraphPad, Inc., La Jolla, CA, USA) software. All samples were assessed for normal distribution using the Shapiro–Wilk test (*p* > 0.05) and QQ plot. The Levene test was used to test homoscedasticity. Two-way ANOVA analysis was carried out as a parametric test. Post hoc comparisons were performed using Fisher’s LSD or Tukey HST tests. The Welch’s *t*-test and Welch ANOVA were performed when the variances demonstrated heterogeneity. The results for normal distribution are presented on the graphs as mean ± standard error of the mean (SEM). When nonparametric statistics were required, the Kruskal–Wallis U test was performed followed by a post hoc Dunn’s test, with the data presented as medians on the graphs. The statistical significance level was set at *p* < 0.05.

## Data Availability

Data are not publicly available due to ethical reasons. Further enquiries should be directed to the corresponding author.
